# Mouse Models for Immunotherapy in Hepatocellular Carcinoma

**DOI:** 10.3390/cancers11111800

**Published:** 2019-11-15

**Authors:** Enya Li, Li Lin, Chia-Wei Chen, Da-Liang Ou

**Affiliations:** 1 Graduate Institute of Oncology, College of Medicine, National Taiwan University, Taipei 100, Taiwan; enyali@gmail.com (E.L.); blackmarketeer61@yahoo.com.tw (L.L.); d06453002@ntu.edu.tw (C.-W.C.); 2 Department of Chemical and Biomolecular Engineering, University of California, Irvine, CA 92697, USA; 3 National Center of Excellence for Clinical Trial and Research, National Taiwan University Hospital, Taipei 100, Taiwan; 4 Department of Oncology, National Taiwan University Cancer Center, Taipei 100, Taiwan

**Keywords:** hepatocellular carcinoma, immunotherapy

## Abstract

Liver cancer is one of the dominant causes of cancer-related mortality, and the survival rate of liver cancer is among the lowest for all cancers. Immunotherapy for hepatocellular carcinoma (HCC) has yielded some encouraging results, but the percentage of patients responding to single-agent therapies remains low. Therefore, potential directions for improved immunotherapies include identifying new immune targets and checkpoints and customizing treatment procedures for individual patients. The development of combination therapies for HCC is also crucial and urgent and, thus, further studies are required. Mice have been utilized in immunotherapy research due to several advantages, for example, being low in cost, having high success rates for inducing tumor growth, and so on. Moreover, immune-competent mice are used in immunotherapy research to clarify the role that the immune system plays in cancer growth. In this review paper, the advantages and disadvantages of mouse models for immunotherapy, the equipment that are used for monitoring HCC, and the cell strains used for inducing HCC are reviewed.

## 1. Introduction

Liver cancer is one of the dominant causes of cancer-related mortality, and the survival rate of liver cancer is among the lowest for all cancers [1]. In 2012, the highest incidences of liver cancer were reported in East Asia and Southeastern Asia [2], and liver cancer is predicted to be the third most lethal cancer by 2023 [3]. Hepatitis virus infection, excessive alcohol, aflatoxin B1 exposure, diabetes, and obesity are the main risk factors correlated with the development of hepatocellular carcinoma (HCC). The occurrence of HCC is generally initiated by necroinflammation that subsequently leads to fibrosis, then cirrhosis, and ultimately to HCC [4]. Developing innovative HCC therapies has been difficult due to the high intrinsic resistance of HCC, damaged functional reserves, and unclear HCC driver genes [5]. As such, the current advanced HCC treatments only slightly increase patient survival [6]. At the same time, immunotherapy has yielded some encouraging results suggesting that new, more effective drugs may soon be developed for this difficult to treat cancer [7].

Sorafenib was first approved by the U.S. Food and Drug Administration (FDA) as a multikinase inhibitor for advanced HCC treatment and was reported to be capable of improving patients’ overall survival in comparison to placebo as a first-line treatment. Nevertheless, sorafenib resistance and side effects are commonly observed. Regorafenib, cabozantinib, ramucirumab, nivolumab, and lenvatinib have also presented positive outcomes in clinical trials, and regorafenib, nivolumab, and ramucirumab are currently suggested as second-line therapy options, whereas lenvatinib has potential as a first-line treatment [8,9,10]. The agents mentioned above inhibit vascular endothelial growth factor (VEGF), but their clinical benefits thus far remain limited [5,11]. Thus, the identification of new immune targets and checkpoints and the customization of treatment procedures for individual patients are among the prospective trends for future advances in immunotherapy [12]. Immune checkpoint inhibitors have demonstrated encouraging therapeutic results in immunotherapy. However, only a small fraction of patients has responded to single-agent therapies. Thus, further research leading to new combination therapies, the development of additional immunotherapeutic drugs, and the improvement of clinical trial success rates is urgently needed [13,14].

Mice are low in cost, have a short reproductive cycle and high tumor growth rate, and are susceptible to genetic manipulation. Inbred strains of mice have been created that allow autologous tumor transplantation from another same strain mice or from the cell line derived from the same strain mice. Those benefits make mouse models a good approach to improve HCC therapy. However, the translatability of discoveries in mouse research to human clinical trials is commonly questioned given the high failure rate observed in human clinical trials subsequent to promising results in mouse studies. Being capable of simulating the microenvironment of human cancer growth genetically, physiologically, and anatomically is vital for the mouse models used for HCC immunotherapy research [15]. Relatedly, immune-competent mice are utilized by immunologists in order to thoroughly understand the function of the immune system in cancer growth [16]. Orthotopic-, diethylnitrosamine, hepatitis B and C virus -induced mouse models are exclusive HCC mouse models.

**Figure 1:**
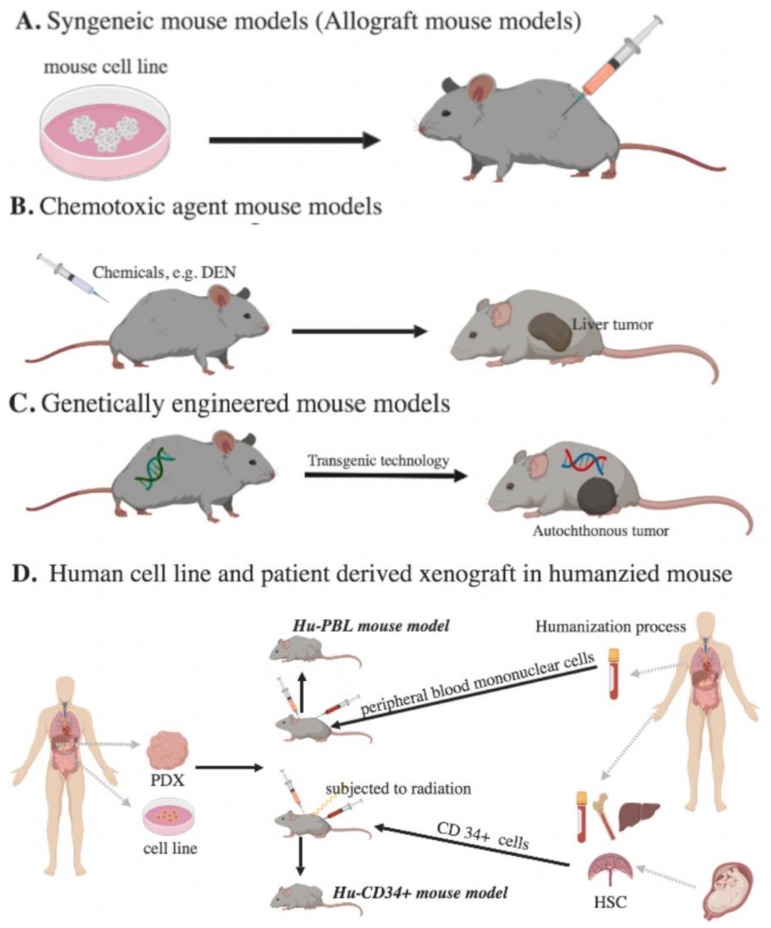
Murine models for immunotherapy studies of HCC. (**A**) Syngeneic mouse models: Mouse tumor cells are implanted in immune-competent mice. (**B**) Chemotoxic agent mouse models: Chemicals are administered to induce HCC growth. (**C**) Genetically engineered mouse models: Tumor suppressor gene deletion or oncogene activation is built into mice. (**D**) Human cell line and patient-derived xenograft in humanized mouse models: Human peripheral blood mononuclear cells (PBMC) or human CD34^+^ cells are given to immunodeficient mice. (PDX, patient-derived xenografts; Hu-PBL, PBMC-humanized mouse model; HSC, hematopoietic stem cells).

**Figure 2:**
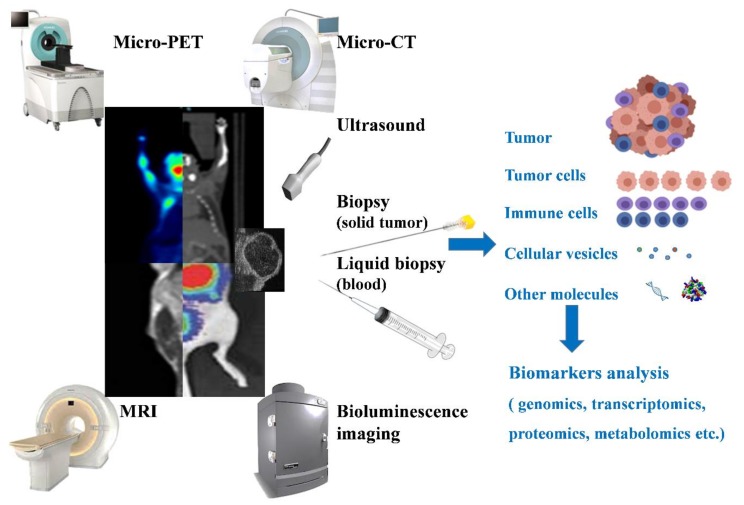
Equipment or methods used for in vivo tumor monitoring. Micro-PET, Micro-CT, MRI, ultrasound, and bioluminescence are the commonly used methods for monitoring tumors non-invasively. Besides the imaging of tumors and surrounding tissues that they can provide, based on their imaging mechanism, other parameters can be provided to determine the status of a tumor. For example, the blood flow and the hypoxic regions can be used to identify the degree of angiogenesis in a tumor. Biopsy (and liquid biopsy) are also methods used for monitoring tumors, with analysis of the cells (tumor or immune cells), proteins, DNA, or any detectable tumor-related marker being used to understand or predict the tumor condition.

Herein, we aim to review different applicable mouse models for immunotherapy (Figure 1), their pros and cons (Table 1), details regarding the cell strains for inducing HCC growth (Table 2), and the equipment that are extensively used for monitoring tumor growth (Figure 2).

## 2. Syngeneic Mouse Models

Syngeneic mouse models, also known as allograft mouse tumor systems, are mouse models that utilize immune-competent mice as the recipient mice for the implantation of mouse tumor cells. Tumor cells can be injected orthotopically or ectopically. Orthotopic mouse models provide more precise tumor microenvironments, whereas subcutaneous mouse models allow for the easy monitoring of and operations on tumors. For HCC induction, tumor cells are injected in the liver for orthotopic models, while ectopic models most commonly receive subcutaneous injections in the flanks [26] (see Table 2).

The benefits of syngeneic mouse models include their fast tumor growth, reproducibility, low cost, ease of tumor progression monitoring, and comparative ease of utilization. A statistically meaningful number of syngeneic mice needed for an experiment can be produced within a short period of time. Thus, syngeneic mouse models are commonly used in survival analysis, observational studies, and experiments that require a large sample size [40]. Another advantage of using syngeneic tumor models is that the tumor cell lines can be generated to express or not express specific biomarkers by vector transferring, which is important for immunotherapy studies in terms of identifying therapy targets and predictive and prognostic factors [34]. However, the complexity and heterogeneity of human HCC might not be completely replicated in such models, and a vaccination effect might be caused due to the existence of dead cancer cells after injection. Other drawbacks are that the tumors develop rapidly rather than growing in a natural setting with chronic inflammation and that the tumors do not develop from natural cells [41]. Moreover, the available selection of mouse cancer cell lines is comparably less diverse than that of human cancer cell lines, and many of the mouse cancer cells were triggered by chemicals [17,42]. In addition, since the time for tumor progression is fast, the experimental window is short [18,19,20,21,22]. At the same time, since important differences between mouse and human immune systems exist in the signaling pathways in T cells, receptor expression in immune cells, and antigen processing and presentation machinery [17,43], various improvements have been made to mouse models to increase their heterogeneity and better reflect human cancer microenvironments. For example, Reiberger et al. [32] utilized tetrachloride for liver cirrhosis induction in an orthotopic mouse model.

C57BL/6J is the colony maintained in the Jackson Laboratory, and C57BL/6N substrain was established in the National Institutes of Health. Many phenotypic differences between C57BL/6J and C57BL/6N substrains have been reported [44]. The wildly used and established HCC cell line, Hepa 1-6, is derived from C57BL/6J mice. Hence, if Hepa 1-6 cell line is transplanted into any mice strain besides C57BL/6J, it cannot be called as a syngeneic mouse model. By utilizing syngeneic mouse models, Chen et al. [45] revealed the improvement of anti-programmed death receptor-1 (PD-1) immunotherapy by CXCR4 inhibition in a sorafenib-treated C3H orthotopic model; Lu et al. [46] demonstrated that exosomes, derived from dendritic cells, can function as a vaccine to promote tumor regression in a C57BL/6 orthotopic model; Li et al. [40] showed that RIP3 deficiency in the liver drives myeloid-derived suppressor cell (MDSC) recruitment in a C57BL/6 orthotopic model; and Ou et al. [47] found that lenalidomide combined with sorafenib demonstrates potential synergistic anti-tumor effects in a BALB/c orthotopic model. In still other studies, Yu et al. [33] reported that MDSC levels were increased in response to cytokine-induced killer cell therapy, a therapy performed by administering a mixture of immune cells to eliminate cancer cells, while Ou et al. [34] reported that PD-L1 can be expressed in an orthotopic model to clarify the role of PD-L1 in various immunotherapies.

## 3. Chemotoxic Agent Mouse Models

The chemicals applied to encourage tumor growth in mouse models of HCC are considered carcinogens [48]. Direct DNA damage or the promotion of preneoplastic cell growth after hepatotoxic compound initiation are the two mechanisms for tumor formation after carcinogen induction. Diethylnitrosamine (N-nitrosodiethylamine, DEN) is a chemotoxic agent that causes DNA damage through the alkylation of DNA or oxidative stress promotion [21,49,50,51]. The liver is mainly targeted by DEN due to its activation by cytochrome P450, an enzyme highly active in the liver. DEN is also able to increase the production of IL-6 in Kupffer cells. The IL-6 trans-signaling in turn promotes angiogenesis and oncogenesis by enhancing tumor proliferation through β-catenin promotion and suppressing apoptosis via p53 inhibition [52,53]. Besides DEN, peroxisome proliferators, aflatoxin B1, CCl_4_, choline deficient diets, and thioacetamide also promote carcinogenicity [26]. Furthermore, Schietinger et al. [31] reported that tamoxifen induced HCC after about 5 months in BALB/c mouse. 

Besides the age, strain, and sex of the mice, the time at which tumor formation is induced by DEN is also associated with the size of the dose administered [54,55]. The gender difference is caused by the inhibition of IL-6 production in Kupffer cells mediated by estrogen [53], and the age difference is due to the high hepatocyte proliferation rate in younger mice [52]. C57BL/6 male mice with an age of 12–15 days are frequently subjected to 25 mg/kg doses of DEN via intraperitoneal injection since cytochrome P450 levels in mice decrease after the 15th day of life. Subsequent analyses, meanwhile, are generally conducted at around 8 months after injection (Table 2). However, human HCC is generally identified in adults, and utilizing adult mice requires an additional tumor-promoting agent and takes around 6–9 months to induce HCC. Combining DEN with other carcinogens can also speed up the tumor formation [29]. The combination of DEN, carbon tetrachloride (CCl_4_), and alcohol, for example, has caused adult mice older than 21 days to develop HCC within five months. 

Chemical-induced models are easy to work with, and the tumors formed after carcinogen induction can better reflect sporadic cancers in humans [19,50]. The HCC developed after DEN treatment in mice has been shown to be similar to human HCC with a poor prognosis in terms of the gene expression [30]. Therefore, DEN-induced models are often utilized in gene profile analysis [46]. However, unlike typical human HCC initiated by chronic inflammation, HCC in mice treated with DEN is induced by serious DNA damage [30].

DEN mouse models have been widely utilized for various research purposes. For example, Lee et al. [23] reported that pIC acts as an inhibitor of HCC initiation through NK cell activation, reprogramming M1 and M2 macrophages in DEN-treated mice. In the immunonutritional field, Laparra et al. [29] reported that serine-type protease inhibitors have the ability to modulate innate immunity and decrease HCC aggressiveness and progression in DEN-treated mice. With regard to new reagent design, Li et al. [56] reported that nanoliposome C6-ceramide, a form of ceramide, increases the anti-tumor immune response in a DEN-induced HCC mouse model. The effects of specific genes on tumor progression can be revealed by giving DEN to mice with gene knockouts or knock-ins. Wang et al. [28] reported that the inactivation of both Akt1 and Akt2 induced rapid HCC formation in a DEN-induced model, and Li et al. [40] reported that the paucity of RIP3 encourages MDSC recruitment within liver tumors by the CXCL1-CXCR2 axis. Another research direction consists of seeking to understand the relationships among cell population dynamic changes within oncogenesis after DEN injection. Shen et al. [57] reported the role of TGF-β in the induction of Treg cell polarization in DEN-induced HCC, and Finkin et al. [36] reported that DNA damage might play a role in the formation of ectopic lymphoid-like structures. Meanwhile, Suk et al. [27] presented the opposing roles of liver fibrosis regulation played by cannabinoid receptors 1 and 2 in hepatocarcinogenesis.

## 4. Genetically Engineered Mouse Models (GEMMs)

Using transgenic technology, GEMMs are built with tumor suppressor gene deletion or oncogene activation, and tumor development is thus induced. Longer tumor development times are required with GEMMs; however, all the stages of cancers can be recapitulated in terms of their genetic and histopathological aspects. Mice with single or multiple gene inactivation and hepatitis B virus (HBV) and hepatitis C virus (HCV) genome activation have previously been reported as the GEMMs used for HCC research [18,19,21]. The over-expression of oncogenes including Myc protein and β-catenin with H-ras genes raises the chance of HCC induction, while the over-expression of growth factors, e.g., transforming growth factor-α, epidermal growth factor, fibroblast growth factor 19, and simian vacuolating virus 40, can also cause tumor development. Mice lacking alpha-1 antitrypsin, phosphatase and tensin homolog, and glycine N-methyltransferase, or overly expressing platelet-derived growth factor and transforming growth factor-beta, also develop HCC [26].

While GEMMs encompass the gene alterations participating in various pathways that are crucial for cancer metastasis or development, the tumors in GEMMs are generally homogenous and do not represent the tumor heterogeneity seen in humans. However, various tumor microenvironments are effectively incorporated in GEMMs. For example, hepatic necrosis, inflammatory infiltration, and periportal fibrosis, stages of liver injury similar to those in humans, are demonstrated in mice with hepatocyte-specific ablation of Shp2 [35]. With albumin-CRE systems, tumor suppressor genes or liver-specific oncogenes of interest can be studied [23,28]. Another commonly used system in inducing HCC is tetracycline-controlled system, a tetracycline-controlled transactivator protein (tTA), driven by the liver-enriched activator protein promoter. tTA expresses in liver only, and a tetracycline-responsive promoter element (TerR) can turn the target gene on or off. In this case, Lai et al. [58] showed a spontaneous HCC mice model that expressed oncogenes, Myc. Chung et al. [59] utilized hydrodynamic transfection to develop a GEMM for mimicking human HCC. The cMyc expression, as well as p53 downregulation resulting from the expression of short hairpin RNA, were observed. In addition, carbon tetrachloride was administered to cause liver fibrosis, a feature generally not shown in other current GEMMs. Liu et al. [60] demonstrated that by delivering naked DNA via hydrodynamic injection into liver cells, HCC can be induced in a mouse model. Based on a transposon system, it is possible to use this model to test the different oncogene functions in several conditions. This transfection makes HCC express bioluminescence and a tumor-specific antigen, and it is convenient for monitoring tumor-specific T cell statuses or metastasis. Also, this model will form diffuse or nodular liver tumors. This might suggest the mechanism of a different type of tumor formation. Multidrug resistance 2 gene (Mdr2)-knockout (Mdr2-KO) mouse model is a well characterized model of chronic liver inflammation, caused by the lack of phospholipids in the bile. The increased biliary concentration of non-micellar-bound free bile acids leads to the damage of the tight junctions of biliary epithelium with the basement membrane. The damage will further cause bile leakage to the portal tract and induce inflammation and fibrosis that eventually followed by HCC [61,62]. The model is a good strategy to induce chronic inflammation-fibrosis-HCC. Barashi et al. [63] and Potikha et al. [64] showed that double knockout mice can identify the important molecules that participated in inflammation-fibrosis-induced hepatocellular carcinoma. Spontaneous metastasis in GEMMs would immensely benefit the field of GEMMs [22]. The current GEMMs have mostly failed to completely mimic the interactions between T cells and tumor antigens [19]. Studies have reported that T cells are unsuccessful at responding to de novo tumors due to tumor-induced T-cell tolerance [65,66,67]. Viral oncogenes playing the role of oncogenic drivers and tumor-specific antigens can be used in studies aimed at improving the understanding of antigen or effector cells [31]. In one study, a constitutively active IKKβ mouse model was reported to form HCC [36]. It has also been demonstrated that a HBV antigen vaccination combined with immune checkpoint blockade induced HCC in HBV antigen transgene mice, which created a suitable approach for studying immunotherapy in HBV-induced HCC mouse models [37].

Besides being utilized for tumor formation, GEMMs can also serve as models for understanding the interactions between tumor antigens and CD8 T cells. For example, Li et al. [68] reported the mechanism of CD8 T cell exhaustion driven by B7 superfamily member 1, and Goel et al. [38] showed that CDK4/6 inhibitors induce tumor regression and increase antigen expression by T cell receptor modified mouse models.

HBV or HCV infection is one of the unique approaches used to induce HCC that cannot be used to induce most other cancers. HBV and HCV are two viruses associated with the development of HCC [69]. It is possible to create a GEMM model through viral product expression in liver cells to simulate a viral infection in the liver, such as through the expression of the surface antigen (HBsAg) or the gene X (HBx) of HBV. HBx was shown to have the ability to bind to p53 to increase proliferation and survival rate of HCC cells by compromising DNA damage checkpoints and activating various growth control genes, such as tyrosine kinases, Ras, Raf, MAPK, ERK, and JNK, in infected liver cells [69].

HBx transgenic mice have been reported to develop HCC at a rate of 80%~90%; however, it takes them 15 to 24 months to do so [53,69]. To speed up the HCC development process, additional carcinogens, such as DEN, should be provided [70]. In other words, HBx expression sensitizes liver cells to carcinogen-induced cancer formation. 

Due to their relatively long tumor progression time, HBx transgenic mice models are utilized as an approach to understand the relationship between HBV infection and HCC and are not the first choice for experiments that do not require viral infection to answer the research question. However, for experiments focused on the mechanisms by which viral products induce HCC [71] and the consequences of their interactions with other oncogenes [72], viral gene transgenic mice models are more suitable.

## 5. Humanized Mouse Models

Humanized mouse models can serve as immunodeficient mice with peripheral blood mononuclear cells (PBMCs) or human CD34^+^ cells injected, or as immunocompetent mice with human gene expressions enabled through transgenesis [14]. Mice with the IL-2rγ^null^ mutation are identified as having impaired T and B cell development, NK cell deficiency, and limited lymph node growth. They allow for hematopoietic cell growth after the transfer of human hematopoietic stem cells (HSCs) [73].

The PBMC-humanized mouse model (Hu-PBL) is appropriate for short-term research and immune function investigation of patients suffering from immune dysfunction since the lymphocytes separated from peripheral blood are mature. The mice can be utilized instantly upon PBMC engraftment [24]. Xenogeneic graft-versus-host-like disease generally occurs in engrafted IL-2rγ^null^ mice after a few weeks and is expedited by subjecting the mice to irradiation before PBMC engraftment. Although only a short investigation window is allowed, human CD3+ T cells can rapidly engraft into the Hu-PBL model within one week [25,73].

The CD34^+^-humanized mouse model (Hu-CD34^+^) supports studies of immune system development since the human stem cells injected in the mouse differentiate into T and B cells under negative selection. At least 10 to 12 weeks are required for HSCs to engraft in mice. Irradiation is required to ensure cell engraftment into the impaired bone marrow [24,74]. HSCs are accessible in granulocyte colony-stimulating factor-mobilized PBMCs, adult bone marrow, fetal liver, and umbilical cord blood [14]. Human umbilical cord blood leads to better engraftment percentages than adult bone marrow, with 4 × 10^4^ umbilical cord blood cells attaining an equal degree of engraftment as 2 × 10^4^ adult bone marrow cells [73]. Newborn mice demonstrate better HSC engraftment than adult mice, and besides the age of mice, the strain and route of engraftment are other variables that control the HSC engraftment ability [75]. The entire human immune system can be established in CD34^+^-humanized mice; however, the process of human unconventional T lineage development cannot be completely simulated since additional human-specific factors are demanded in the murine thymus [72]. The deficiency in human Foxp3^+^ T cell formation in humanized mice was observed to be the most noteworthy difference between human and murine thymuses [76].

Patient-derived xenografts (PDXs) are also important mouse models in the study of HCC. The lack of an accurate reflection of the heterogeneity in tumors is always one of the primary disadvantages of syngeneic mouse models and GEMMs, and the implantation of tumors derived directly from patients is one way to solve this problem [39]. Moreover, the given graft can come from a surgical resection or biopsy, making it suitable for stage-related experiments [77]. However, without the human immune system, PDX mouse models may not be able to precisely represent the status of tumors [20]. Therefore, it is only by combining the humanized mouse and the PDX model to form a PDX in humanized mouse model that both of their limitations can be overcome, resulting in a mouse model with a tumor derived from patient that also has a human immune system to interact with. A humanized liver with human immune system mouse model could further advance the degree to which the model accurately reflects HCC in humans [78], and the ability to create ectopic livers in mice might even provide greater feasibility for the study of tumor microenvironments [79].

## 6. Equipment for In Vivo Tumor Monitoring

Various methods can be utilized to track the conditions of tumors in mouse models. Micro-computed tomography (micro-CT), micro-positron emission tomography (micro-PET), and nuclear magnetic resonance imaging (MRI) are three methods that are widely used for acquiring tumor images in patients, and they can also be utilized in mouse models. These techniques can provide a full view of a tumor and its surrounding tissue. However, there are drawbacks to their use. Mice that undergo micro-CT and micro-PET imaging are exposed to radioactive tracers, treatment results might be affected, and all three approaches are time-consuming to perform [80].

Ultrasound is also commonly used for monitoring the conditions of tumors. It is cheaper in cost than the aforementioned methods, and the imaging can be performed in real-time while other experimental procedures are being conducted simultaneously. Despite lacking the detailed information that micro-CT/PET or MRI can provide, the high soft-tissue contrast of ultrasound is sufficient to locate the tumor for injection or surgery. Bioluminescence imaging is useful, meanwhile, for non-invasively detecting the spread pattern of an implanted tumor. However, it requires tumor cell lines that have already transfected the luciferase gene, and it might underestimate the size of a tumor at the late stage because of the decline of bioluminescence reaction that is caused by the hypoxia status occurring in the tumor which decreases the ATP and oxygen partial pressure level [81].

In vivo tumor imaging is not sufficient for immunotherapy research or diagnosis. Immune signatures for cancer, such as immune cell profiles, the immune-related markers expressed, and DNA, RNA, and tumor or immune cells that travel within the blood stream, can provide vast information for immunotherapy study. Based on the principles of PET and MRI imaging, it is possible to connect an antibody to a specific immune-related marker with a magnetic agent or radionuclide [82]. In this way, the immune-related markers can be detected with the same equipment and maintain their benefits. Natarajan et al. [83] demonstrated that a ^64^Cu-labeled anti-mouse PD-1 antibody was detected by PET, providing the potential ability to evaluate the prognostic marker in mouse models and immune checkpoints in related research. Han et al. [84] further improved upon this method by creating a tag-antibody-drug complex, which can directly show the effectiveness of drug delivery and therapy.

Besides in vivo tumor imaging techniques, non-imaging tests are also crucial for monitoring the statuses of tumors. Blood flow is an important factor for determining ongoing angiogenesis. The effects of a treatment can be evaluated by detecting the performance of blood vessels, which can be easily done by ultrasound. MRI can detect the blood oxygenation level-dependent contrast change within a tumor, meaning that the degree of hypoxia, a significant factor in tumor progression, can also be calculated [85]. Other immune signatures can be detected by monitoring methods such as flow cytometry or mass cytometry, and fluorescent or metal-mass based antibodies are utilized to identify specific markers [86,87]. Single-cell RNA sequencing can also provide information about immune signatures [88], and the greater the number of cell subtypes that are identified, the better the understanding of immunotherapy that can be achieved. Epigenomic profiling can be tissue- and status-specific, making it suitable for monitoring tumor profile changes and distinguishing a tumor from normal tissues [89]. All the non-imaging techniques reviewed above require biopsy procedures. The liquid biopsy process is much easier than the process for traditional biopsies since only blood is required for the former process, and a great amount of bio-markers can be examined in cells, cell-free DNA, or RNA and exosomes. Cell-free DNA can provide a vast amount of information [90], but its representative ability has been questioned, with the answers to a number of specific questions remaining unclear. For example, should the liquid biopsy sample come from the tumor? When is the appropriate time to perform a liquid biopsy after a treatment? Does the change in substance concentration relate to treatment, or are there other unknown factors? Are there any other informative substances that are neglected? To answer whether the response information gathered from a liquid biopsy is associated with a therapy or other factors, the answers to the questions above have to be clarified first. There is no single method, however, that can be used to solve all of the associated problems. Thus, combining techniques is a good way to overcome the limitation of some methods and to further understand the mechanisms of immunotherapy.

## 7. Conclusions and Future Perspectives

The advent of immune checkpoint inhibitors and adaptive cell therapies has revolutionized cancer therapy, and associated preliminary trials regarding HCC have shown encouraging results.

The mouse models used in related research have various pros and cons. In order to identify the appropriate model for a given study, one should determine exactly what experimental questions one is seeking to answer. To improve the heterogeneity and microenvironment of a syngeneic mouse model, various cancer cell lines can be injected into the model; for example, Calbo et al. [91] injected various small cell lung cancer cells.

The shorter the amount of time required for a protocol to result in tumor development, the higher the death rate among the mice subjected to the protocol. However, adjusting the dosing by monitoring the features of the mice, such as body weight loss, might be one way to prevent mice from dying unnecessarily.

As noted in the preceding sections of this review, there are several advantages and disadvantages to the various models that can be chosen as the model for inducing HCC in mice. Through unique mechanisms, tumors can be induced in mice in ways that allow researchers to choose from different time scales of progression, lesion sites, and tumor microenvironments. Although the different models have different profiles, analyzing their shared features might yield many clues to help researchers better understand the mechanisms underlying HCC formation.

## Figures and Tables

**Table 1:** The advantages and disadvantages of murine models for immunotherapy studies of hepatocellular carcinoma (HCC).

Mouse Model		Advantages	Disadvantages	Reference
Syngeneic		• Not artificial	• Lack of diverse cancer cell line and heterogeneity	[15,17,18,19,20,21,22]
		• Easy to utilize	• Mostly chemical induced cancer cell line	
		• Rapid tumor development	• Tumor does not develop from normal cells or develop in a natural microenvironment	
		• Reproducible	• Mouse and human immune systems have vital differences	
		• Low in cost	• Might cause a vaccination effect	
		• Tumor can be accurately monitored without difficulty	• Human HCC cannot be completely recapitulated by mouse cancer cells in terms of the complexity, histology, and natural carcinogenesis characteristics	
		• Non-immunogenic	• Short experimental window	
Chemotoxic agent		• Easy to work with	• Tumor formation is not initiated by chronic inflammation	[18,19,20,21,23]
		• Sporadic cancer development		
		• Higher heterogeneity	• Difficult to monitor a tumor	
		• Tumors generally progress in a natural microenvironment and develop from normal cells	• Variability in the time for tumor progression	
		• Available to incorporate with other approaches for tumor induction	• Larger sample sizes are needed for data interpretation due to the high heterogeneity	
GEMM		• Encompasses natural tumor microenvironments	• Longer latency and time for tumor development	[15,17,18,19,20,21,22]
		• The genetic and histopathological aspects of all stages of cancer can be recapitulated	• Difficult to monitor a tumor	
			• Low immunogenicity	
			• Costly and challenging for breeding and gene manipulation	
		• Tumors develop from normal cells	• Homogeneous in the genomic aspect	
Humanized	CD34^+^	• Immediately available for experiment	• 4–8 weeks of experimental window	[15,21,24,25]
		• The complex human immune system and human HCC can be recapitulated	• Difficult to set up	
	PBL	• The entire complex human immune system can be established	• 10-12 weeks are required for HSC engraftment	[15,21,24,25]
			• Difficult to set up	
		• Human HCC can be recapitulated	• High in cost	

**Table 2:** Mouse models used for immunotherapy in HCC. (CA, chemotoxic agent mouse model; SG, syngeneic mouse model; GEMM, genetically engineered mouse model; HMM, humanized mouse model; DEN, diethylnitrosamine).

Model	Growth Site	Mice Background	Inducer	Dose	Tumor Harvest	References
**CA**	Orthotopic	C57BL/6	DEN	10~35 mg/kg/once	8~12 months	[23,26,27,28]
			DEN+ thioacetamide	20 mg/kg/weekly	2 months	[29]
			DEN+ carbon tetrachloride	8 mL/kg/twice a week	6 months	[30]
		BALB/c	Tamoxifen	1 mg/mice/once	4~5 months	[31]
**SG**	Orthotopic	C3H; C57BL/6	HCA-1; RIL-175	1 × 10^6^; 1 × 10^5^	No data; 3 weeks	[32,33]
		BALB/c; C57BL/6	Hepa1 -6; BNL-1MEA	2 × 10^6^	1 week	[26,32,34]
	Subcutaneous	C57BL/6	RIL-175	1 × 10^6^	150 mm^3^	[33]
		BALB/c	BNL-1MEA	1 × 10^6^	200 mm^3^	[33]
**GEMM**	Orthotopic	C57BL/6	Alb-cre Pten		9 months	[26]
			Alb-cre Shp2		2 months	[35]
			Akt1^−/−^,Akt2 ^−/−^		5~6 months	[23]
			Alb-IKKβ		20 months	[36]
			Alb-HBV		28 weeks	[37]
		BALB/c	Alb-floxStop-SV40		7(20) weeks	[31]
		C57BL/6	P14			[38]
**HMM**	Subcutaneous	NSG	Patient-derived tumor		8~10 weeks	[39]
